# Predominance of Abdominal Visceral Adipose Tissue Reflects the Presence of Aortic Valve Calcification

**DOI:** 10.1155/2016/2174657

**Published:** 2016-01-24

**Authors:** Masayoshi Oikawa, Takashi Owada, Hiroyuki Yamauchi, Tomofumi Misaka, Hirofumi Machii, Takayoshi Yamaki, Koichi Sugimoto, Hiroyuki Kunii, Kazuhiko Nakazato, Hitoshi Suzuki, Shu-ichi Saitoh, Yasuchika Takeishi

**Affiliations:** Department of Cardiology and Hematology, Fukushima Medical University, 1 Hikarigaoka, Fukushima 960-1295, Japan

## Abstract

*Background*. Aortic valve calcification (AVC) is a common feature of aging and is related to coronary artery disease. Although abdominal visceral adipose tissue (VAT) plays fundamental roles in coronary artery disease, the relationship between abdominal VAT and AVC is not fully understood.* Methods*. We investigated 259 patients who underwent cardiac and abdominal computed tomography (CT). AVC was defined as calcified lesion on the aortic valve by CT. %abdominal VAT was calculated as abdominal VAT area/total adipose tissue area.* Results*. AVC was detected in 75 patients, and these patients showed higher %abdominal VAT (44% versus 38%, *p* < 0.05) compared to those without AVC. When the cutoff value of %abdominal VAT was set at 40.9%, the area under the curve to diagnose AVC was 0.626. Multivariable logistic regression analysis showed that age (OR 1.120, 95% CI 1.078–1.168, *p* < 0.01), diabetes (OR 2.587, 95% CI 1.323–5.130, *p* < 0.01), and %abdominal VAT (OR 1.032, 95% CI 1.003–1.065, *p* < 0.05) were independent risk factors for AVC. The net reclassification improvement value for detecting AVC was increased when %abdominal VAT was added to the model: 0.5093 (95% CI 0.2489–0.7697, *p* < 0.01).* Conclusion*. We determined that predominance of VAT is associated with AVC.

## 1. Introduction

Aortic valve calcification (AVC) is a common feature of aging and is considered as atherosclerotic changes of the aortic valve [1]. Because early-stage AVC does not present any symptoms, many cases are incidentally diagnosed by cardiac imaging examination for other cardiac or pulmonary diseases. Although the rate of progression of AVC to clinical aortic stenosis is low, AVC is independently associated with cardiovascular mortality [2, 3]. Traditional cardiovascular risk factors are associated with the development of AVC, and obesity is also related to its progression [1]. Central obesity is an indispensable factor for the diagnosis of metabolic syndrome, which is related to the progression of not only coronary artery disease but also AVC [4]. Abdominal visceral adipose tissue (VAT) produces various inflammatory cytokines that cause the development of insulin resistance, which is a fundamental characteristic of metabolic syndrome [5]. Although VAT has been reported to be associated with coronary artery calcification and abdominal aorta calcification [6, 7], few reports have focused on AVC. The purpose of this study was to evaluate the importance of abdominal VAT in the development of AVC.

## 2. Materials and Methods

### 2.1. Subjects

We enrolled 259 patients who had undergone 64-slice computed tomography (CT) angiography for the diagnosis of coronary artery disease from January 2013 through December 2014. Hypertension was defined as the recent use of antihypertensive drugs, systolic blood pressure ≥ 140 mmHg, and/or diastolic pressure ≥ 90 mmHg. Diabetes was defined as the recent use of insulin or antidiabetic drugs, fasting blood glucose ≥ 126 mg/dL, and/or hemoglobin A_1c_ ≥ 6.5%. Dyslipidemia was defined as the recent use of cholesterol-lowering drugs, triglyceride ≥ 150 mg/dL, low-density lipoprotein cholesterol ≥ 140 mg/dL, and/or high-density lipoprotein cholesterol ≤ 40 mg/dL. Patients who had poor image data or a history of open heart surgery or acute coronary syndrome were excluded. The study protocol was approved by the institutional ethics committee, and informed consent was obtained from all study subjects.

### 2.2. CT Scan Protocol

CT examinations were performed using a 64-slice CT scanner (Aquilion 64, Toshiba Medical Systems Co., Ltd., Tochigi, Japan) with a collimation of 64 × 0.5 mm, pixel size of 0.39 × 0.39 mm, gantry rotation time of 350 msec, and tube voltage of 120 kV. The protocol of premedication and CT scan have been described previously [8]. A noncontrast scan was performed at the level of the umbilicus in order to assess the abdominal VAT area. Only one slice at the umbilical level was captured for the sake of reducing radiation exposure. The reconstructed image data were analyzed by a computer workstation (Ziostation2, Ziosoft Inc., Tokyo, Japan).

### 2.3. Evaluation of CT Findings

The areas of abdominal VAT and subcutaneous adipose tissue were measured by an application software (Fat Measurement, Toshiba Medical Systems Co., Ltd., Tochigi, Japan) as previously described [8]. %abdominal VAT area was calculated as the ratio of abdominal VAT area to the total adipose tissue area and multiplied by 100. Epicardial adipose tissue (EAT) area was measured at the level of four-chamber view of the heart. EAT was defined as any tissues presenting between −230 Hounsfield unit (HU) and −30 HU enclosed by the visceral pericardium [8]. Coronary arteriosclerosis was determined as any visible coronary calcification or atheromatous plaque on a coronary artery. We defined AVC as any visible structures presenting more than 130 HU detected on the aortic valve.

### 2.4. Statistics

Data are expressed as mean with standard deviation. When the data were not normally distributed, median with interquartile ranges was reported. Student's *t*-test was used for normally distributed data, and the Mann-Whitney test was used for unequally distributed data. Logistic regression analysis was performed on anthropometric and clinical variables to identify correlates of AVC. The variables of *p* < 0.10 in the univariable logistic analysis were analyzed by the multivariable analysis. The improvement by adding %abdominal VAT to discrimination and net reclassification of risks was assessed by comparing the area under the curve (AUC) of receiver operator characteristics and the estimation of both integrated discrimination improvement (IDI) and net reclassification improvement (NRI) [9]. All tests were two-tailed and *p* values <0.05 were considered statistically significant. All statistical analyses were performed using R software packages version 3.1.2 (R Core Team 2014, Vienna, Austria).

## 3. Results

**Figure 1:**
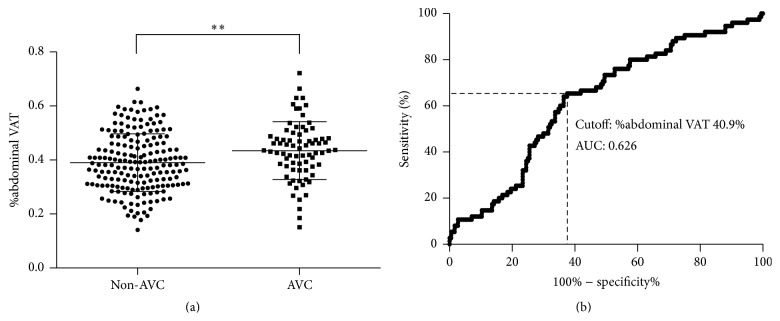
Relationship between %abdominal VAT and AVC. (a) Difference of %abdominal VAT between the non-AVC and AVC groups. Each point represents %abdominal VAT area of the patients. Error bars; mean with standard deviation. ^*∗∗*^ *p* < 0.01 versus non-AVC. (b) ROC curve analysis for the detection of AVC. AUC was calculated when the cutoff value of %abdominal VAT was set at 40.9%.

Patient characteristics are displayed in Table 1. Out of 259 patients, 75 were found to have AVC. Compared to the patients with non-AVC, those with AVC were older and had a higher prevalence of diabetes. Consistent with previous reports, the prevalence of coronary arteriosclerosis was higher in the AVC group. Although the size of abdominal VAT areas was similar between the AVC and non-AVC groups (Table 1), the %abdominal VAT area was larger in the AVC group (Figure 1(a)), suggesting the predominance of abdominal visceral fat in the AVC group.

Figure 1(b) shows a receiver operator characteristic curve for the detection of AVC using a variable of %abdominal VAT. When the cutoff value was determined as 40.9%, diagnostic accuracy was as follows: sensitivity 0.653, specificity 0.625, positive predictive value 0.184, negative predictive value 0.585, and AUC 0.626.

**Figure 2:**
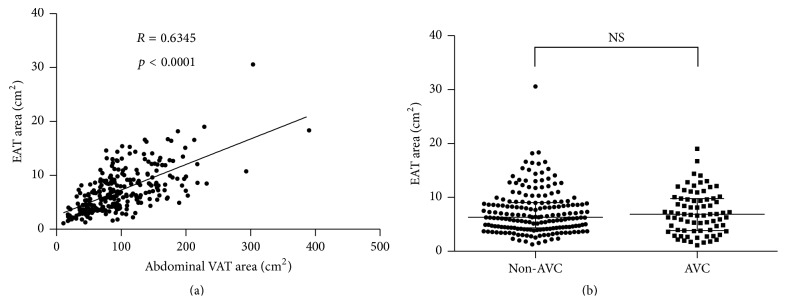
Relationship between EAT and AVC. (a) Correlation between abdominal VAT and EAT. Each point represents abdominal VAT and EAT area of the patients. (b) Difference of EAT area between the non-AVC and AVC groups. Each point represents EAT area of the patients. Error bars; median with interquartile ranges.

Because EAT was also categorized as ectopic visceral adipose tissue and was related to the development of coronary arteriosclerosis [8, 10], we next measured EAT area. As expected, there was a positive correlation between EAT area and abdominal VAT area (Figure 2(a)). Unlike abdominal VAT, there was no difference in EAT area between the presence and absence of AVC (Figure 2(b)).

As shown in Table 2, univariable logistic regression analysis revealed that age, diabetes, and %abdominal VAT were associated with AVC. Multivariable logistic regression analysis indicated that age, diabetes, and %abdominal VAT were independent variables for predicting AVC.

Although it did not reach statistical significance, value of AUC was increased by 1.1% (*p* = 0.25) when %abdominal VAT was added to the model. To assess the importance of adding %abdominal VAT more precisely, NRI and IDI were calculated using the variables with or without %abdominal VAT. The values of NRI for detecting AVC reached statistical significance when %abdominal VAT was added to the model: 0.5093 (95% CI 0.2489–0.7697, *p* < 0.001) for NRI and 0.0116 (95% CI −0.0016–0.0347, *p* = 0.07) for IDI.

## 4. Discussion

In the present study, we demonstrated that increased %abdominal VAT is associated with the presence of AVC. In addition, %abdominal VAT was an independent risk factor of the presence of AVC, and the value of NRI and IDI supported the significance of %abdominal VAT in the development of AVC.

### 4.1. Association of Abdominal VAT and AVC

Several reports showed that oxidative stress, arising from an increased production of free radicals and decreased antioxidant system, is an initial causative stimulus for AVC [1]. Superoxide dismutase (SOD) activity and expression of SOD isoforms were reduced in calcified lesions of human aortic valves [11]. Superoxide was present more abundantly in stenosed aortic valves of hypercholesterolemic mouse hearts [12]. Furthermore, not only reactive oxygen species (ROS) but also inflammatory cytokines are associated with AVC. In patients with aortic valve sclerosis, interleukin-1*β* was upregulated in leukocytes that had infiltrated to the calcified aortic valve [13]. There is a growing body of evidence showing that adipose tissues are related to the production of both oxidative stress and inflammatory cytokines. In obese mice model, ROS were increased with augmented expression of nicotinamide adenine dinucleotide phosphate (NADPH) oxidase, tumor necrosis factor-*α*, and plasminogen activator inhibitor-1 in accumulated adipose tissues [14]. Similarly, nondiabetic obese human subjects showed increased ROS production accompanied with an increase in NADPH oxidase activity [14]. Although we did not have the data regarding the oxidative stress and inflammatory cytokines in this study, these results indicate that increased adipose tissues may contribute to the development of AVC by inducing oxidative stress and inflammation.

In the present study, however, a simple measurement of the abdominal VAT area was similar between the AVC and non-AVC groups. This apparent inconsistency may be explained by the predominance of abdominal VAT. The amount of VAT tends to increase in correlation with total adipose tissue volume, but the proportion of subcutaneous adipose tissue (SAT) and VAT differs from person to person. When energy intake surpasses calorie expenditure, SAT stores excess energy by recruiting new precursor cells, leading to hyperplastic obesity [15]. This type of subcutaneous obesity is characterized by low ectopic fat volume and a normal metabolic profile [16]. However, once recruitment ability of the precursor cells is impaired, adipocyte hypertrophy occurs, and excess energy is stored as ectopic fat [16]. Increased ectopic fat depot, typically abdominal VAT, is strongly related to insulin resistance, producing inflammatory cytokines, and the development of type II diabetes mellitus [16]. Because %abdominal VAT area reflected both SAT and VAT, it may be more feasible parameter than simple assessment of abdominal VAT area. Consistent with this concept, the multivariable logistic analysis showed %abdominal VAT was an independent risk factor of AVC. Moreover, the value of NRI reached statistical significance, indicating the close relationship between %abdominal VAT and AVC. Not only abdominal VAT but also EAT is associated with arteriosclerosis. In the present study, there was no difference in EAT area between the presence and absence of AVC. The mechanisms by which EAT promotes coronary artery disease are partly explained by a direct exposure of inflammatory cytokines produced by EAT to coronary vessel wall [17, 18]. From its viewpoint, aortic valve is apart from EAT, and the volume of EAT is much less compared to abdominal visceral fat. Therefore, we speculate that EAT showed no relationship with presence of AVC. Given that AVC is associated with cardiovascular mortality in spite of hemodynamic insignificance, the presence of AVC may exhibit an early sign of metabolic disturbance affecting the cardiovascular system. Thus, we should take into consideration a detailed assessment of the patient's metabolic profile as well as proper medical management in cases where AVC is detected.

### 4.2. Study Limitations

First, our results are based on a relatively small number of patients and were obtained after excluding lesions with inadequate image quality from analysis. Second, the patients included in the study might have possessed different clinical backgrounds from normal subjects, as they have symptoms or clinical features of suspected coronary artery disease. This raises the possibility that they were likely to be more exposed to arteriosclerotic risk factors than the normal population. Third, Agatston score is useful to classify the severity of aortic valve calcification, but we could not calculate the score because nonenhanced CT was not performed before coronary CT angiography. The relationship between the amount of VAT and severity of AVC remains to be elucidated in the future study. Fourth, the growing body of evidence suggested that both oxidative stress and inflammatory cytokines play important roles in initiating aortic valve calcification. However, unfortunately, the present study was not able to exhibit the direct evidence of the induction of oxidative stress and production of inflammatory cytokines because no data for C-reactive protein, sedimentation rate, myeloperoxidase, and so forth were available. This point should be further examined in the future study.

## 5. Conclusions

The present study revealed the relationship between the presence of AVC and abdominal visceral adiposity. Given the importance of AVC for cardiovascular mortality, we should consider precise assessment of metabolic profile and medical management when AVC is detected.

## Figures and Tables

**Table 1:** Patient characteristics.

	Non-AVC (*n* = 184)	AVC (*n* = 75)
Age (years)	64 ± 12	75 ± 8^*∗∗*^
BMI (kg/m^2^)	24.6 ± 3.9	23.7 ± 4.4
Male (*n*, %)	113, 61%	44, 59%
Hypertension (*n*, %)	106, 58%	53, 71%
Dyslipidemia (*n*, %)	96, 52%	36, 48%
Diabetes (*n*, %)	41, 22%	31, 41%^*∗∗*^
Smoking history (*n*, %)	72, 39%	32, 43%
Abdominal VAT area (cm^2^)	89.7 [67.8–127.0]	94.1 [50.0–138.7]
Subcutaneous adipose tissue area (cm^2^)	110.0 [24.7–151.1]	80.8 [7.4–115.0]^*∗∗*^
Total adipose tissue area (cm^2^)	191.2 [45.2–254.9]	140.0 [18.3–204.4]^*∗∗*^
Coronary arteriosclerosis (*n*, %)	111, 60%	66, 88%^*∗∗*^

Values are expressed as median with interquartile ranges.

AVC, aortic valve calcification; BMI, body mass index; VAT, visceral adipose tissue; ^*∗∗*^ *p* < 0.01 versus non-AVC group.

**Table 2:** Association with the presence of aortic valve calcification.

	Univariate		Multivariate	
	OR (95% CI)	*p* value	OR (95% CI)	*p* value
Age, per 1-year increase	1.119 (1.081–1.164)	<0.001	1.120 (1.078–1.168)	<0.001
BMI, per 1 kg/m^2^ increase	0.939 (0.872–1.007)	0.085	0.968 (0.889–1.050)	0.446
Male	0.892 (0.517–1.549)	0.681		
Hypertension	1.772 (1.006–3.201)	0.052	1.124 (0.580–2.200)	0.730
Dyslipidemia	0.846 (0.493–1.448)	0.542		
Diabetes	2.457 (1.379–4.379)	0.002	2.587 (1.323–5.130)	0.006
Smoking history	1.158 (0.669–1.994)	0.599		
%abdominal VAT, per 1% increase	1.039 (1.013–1.067)	0.003	1.032 (1.003–1.065)	0.033

OR, odds ratio; CI, confidence interval; BMI, body mass index; VAT, visceral adipose tissue.

## Abbreviations

AVC: Aortic valve calcification
VAT: Visceral adipose tissue
CT: Computed tomography
EAT: Epicardial adipose tissue
HU: Hounsfield unit
AUC: Area under the curve
IDI: Integrated discrimination improvement
NRI: Net reclassification improvement
SOD: Superoxide dismutase
ROS: Reactive oxygen species
NADPH: Nicotinamide adenine dinucleotide phosphate
SAT: Subcutaneous adipose tissue.
